# Supplementary material for: Prevalence of depressive symptoms and their determinants among adolescents in Can Tho City, Vietnam: A cross-sectional study
Source: Glob Ment Health (Camb). 2025 Dec 1;12:e139. doi: 10.1017/gmh.2025.10096 (PMC12720387; doi:10.1017/gmh.2025.10096)
Supplement: Tran et al. supplementary material [file S2054425125100964sup001.docx]

**SUPPLEMENTARY FILE**

**S1**

*Results for bivariate independent t-test or one-way ANOVA of categorical variables on CESDR-V scores*

| Variables | MD (95%CI) | t/ F-value | p-value |
| --- | --- | --- | --- |
| Gender | -4.37 (-6.02 to -2.72) | -5.22 | <0.001 |
| Chronic physical condition |  | 16.2 | <0.001 |
| Yes | -5.01 (-10.2 to 0.14) |  |  |
| No | Reference |  |  |
| Do not know | -6.93 (-9.96 to -3.89) |  |  |
| Participation in sport activities |  | 2.64 | 0.049 |
| 0 days | Reference |  |  |
| 1 day | 2.84 (-0.24 to 5.91) |  |  |
| 2 days | 2.97 (-0.30 to 6.25) |  |  |
| More than 3 days | 2.39 (-0.55 to 5.33) |  |  |
| Alcohol consumption | -6.28 (-8.46 to -4.09) | 5.64 | <0.001 |
| Smoke cigarettes | -14.9 (-21.9 to -8.03) | 4.73 | <0.001 |
| Smoke e-cigarettes | -4.70 (-9.54 to 0.15) | 1.90 | 0.058 |
| Smoke shisha | -7.00 (-26.1 to 12.1) | 0.718 | 0.473 |
| Family history of depression |  | 63.9 | <0.001 |
| Yes | -10.36 (-14.4 to -6.31) |  |  |
| No | Reference |  |  |
| Do not know | -13.5 (-16.6 to -10.3) |  |  |
| Experience of serious injury | -3.43 (-5.29 to -1.56) | 3.61 | <0.001 |
| Experience of sexual violence |  | 6.19 | 0.002 |
| None | Reference |  |  |
| Once | 7.18 (2.05 to 12.3) |  |  |
| More than once | -3.12 (-22.3 to 16.1) |  |  |
| Experience of intimate partner violence |  | 5.83 | 0.003 |
| No romantic partner | Reference |  |  |
| Yes | -10.8 (-19.9 to -1.91) |  |  |
| No | -9.32 (-20.7 to 2.10) |  |  |
| Experience of technology-facilitated sexual violence | -4.14 (-5.89 to -2.38) | 4.61 | <0.001 |
| Experience of forced sex | -7.05 (-12.1 to -1.97) | 2.72 | 0.007 |
| Experience of loss of a loved one | 2.26 (0.598 to 3.92) | 2.67 | 0.008 |
| Experience of school bullying | -7.34 (-10.6 to -4.07) | 4.45 | <0.001 |
| Experience of cyber bullying | -6.98 (-10.7 to -3.29) | 3.75 | <0.001 |
| Monthly household income per capita |  | 3.36 | 0.035 |
| Low | -1.62 (-6.75 to 3.50) |  |  |
| Middle | -2.23 (-4.26 to -0.20) |  |  |
| High | Reference |  |  |
| Experienced domestic physical abuse involving beating | 8.99 (6.17 to 11.8) | 6.30 | <0.001 |
| Experienced domestic physical abuse involving weapon | 10.2 (6.24 to 14.2) | 5.10 | <0.001 |
| Experienced domestic emotional abuse involving verbal insults | 8.95 (6.73 to 11.2) | 7.94 | <0.001 |
| Experienced domestic emotional abuse involving neglect | 10.6 (7.88 to 13.4) | 7.66 | <0.001 |
| Living with a family member who abuses substances | 3.97 (1.56 to 6.38) | 3.22 | 0.001 |
| Living with a family member who has been to prison before | 6.72 (3.09 to 10.4) | 3.63 | <0.001 |
| Witnessed verbal abuse at home | 8.80 (6.96 to 10.6) | 9.40 | <0.001 |
| Witnessed physical abuse at home involving beating | 9.20 (6.70 to 11.7) | 7.24 | <0.001 |
| Witnessed physical abuse at home involving weapon | 8.04 (4.92 to 11.2) | 5.09 | <0.001 |
| Separated or divorced parents | 4.02 (0.994 to 7.05) | 2.63 | 0.01 |
| Phone ownership | 3.68 (-1.69 to 9.04) | 1.35 | 0.179 |
| Family rules on phone use | -0.242 (2.24 to 1.78) | -0.237 | 0.812 |

**S2**

*Correlation coefficients for Pearson’s two-tailed tests*

| Variables | CESDR-V | 95% CI |
| --- | --- | --- |
| Age | -0.046 (p=0.139) | -0.106 to 0.015 |
| TMI | -0.012 (p=0.704) | -0.072 to 0.049 |
| Frequency of physical activity per week | -0.125*** (p<0.001) | -0.184 to -0.065 |
| EVES | -0.002 (p=0.958) | -0.062 to 0.059 |
| CCI | 0.679*** (p<0.001) | 0.645 to 0.710 |
| SCS | -0.178*** (p<0.001) | -0.236 to -0.119 |
| ESSA | 0.516*** (p<0.001) | 0.470 to 0.559 |
| Perceived parental involvement (Homework) | -0.087** (p=0.004) | -0.147 to -0.027 |
| Perceived parental involvement (Understanding) | -0.297*** (p<0.001) | -0.351 to -0.241 |
| Perceived parental involvement (Hobbies) | -0.207*** (p<0.001) | -0.264 to -0.148 |
| Perceived parental involvement (Advice) | -0.281*** (p<0.001) | -0.336 to -0.224 |
| Perceived parental involvement (Expectations) | 0.092** (p=0.003) | 0.032 to 0.152 |
| Duration of internet use | 0.174*** (p<0.001) | 0.114 to 0.231 |
| Duration of screen time | 0.165*** (p<0.001) | 0.106 to 0.223 |
| Duration of online gaming | 0.095** (p=0.002) | 0.035 to 0.155 |

Note: * indicates that p <0.05; ** indicates that p<0.01 and *** indicates that p<0.001. CESDR-V stands for Center for Epidemiologic Studies Depression Scale Revised – Vietnamese Version; TMI stands for Tri-ponderal Mass Index; EVES stands for Self-esteem Scale for Vietnamese adolescents; CCI stands for Crandell Cognitions Inventory (Short Form); SCS stands for School Connectedness Scale; ESSA stands for Educational Stress Scale for Adolescents.
